# Computed Tomography Based Score of Early Ischemic Changes Predicts Malignant Infarction

**DOI:** 10.3389/fneur.2021.669828

**Published:** 2021-06-07

**Authors:** Matthias Bechstein, Lukas Meyer, Silke Breuel, Tobias D. Faizy, Uta Hanning, Noel van Horn, Rosalie McDonough, Jens Fiehler, Gabriel Broocks

**Affiliations:** Department of Diagnostic and Interventional Neuroradiology, University Medical Center Hamburg-Eppendorf, Hamburg, Germany

**Keywords:** stroke, brain herniation, biomarkers, computerized tomography, malignant infarction, edema quantification

## Abstract

**Background and Purpose:** Identification of ischemic stroke patients at high risk of developing life-threatening malignant infarction at an early stage is critical to consider more rigorous monitoring and further therapeutic measures. We hypothesized that a score consisting of simple measurements of visually evident ischemic changes in non-enhanced CT (NEMMI score) predicts malignant middle cerebral artery (MCA) infarctions (MMI) with similar diagnostic power compared to other baseline clinical and imaging parameters.

**Methods:** One hundred and nine patients with acute proximal MCA occlusion were included. Fifteen (13.8%) patients developed MMI. NEMMI score was defined using the sum of the maximum diameter (anterior-posterior plus medio-lateral) of the hypoattenuated lesion in baseline-CT multiplied by a hypoattenuation factor (3-point visual grading in non-enhanced CT, no/subtle/clear hypoattenuation = 1/2/3). Receiver operating characteristic (ROC) curve analysis and multivariable logistic regression analysis were used to calculate the predictive values of the NEMMI score, baseline clinical and other imaging parameters.

**Results:** The median NEMMI score at baseline was 13.6 (IQR: 11.6–31.1) for MMI patients, and 7.7 (IQR: 3.9–11.2) for patients with non-malignant infarctions (*p* < 0.0001). Based on ROC curve analysis, a NEMMI score >10.5 identified MMI with good discriminative power (AUC: 0.84, sensitivity/specificity: 93.3/70.7%), which was higher compared to age (AUC: 0.76), NIHSS (AUC: 0.61), or ischemic core volume (AUC: 0.80). In multivariable logistic regression analysis, NEMMI score was significantly and independently associated with MMI (OR: 1.33, 95%CI: 1.13–1.56, *p* < 0.001), adjusted for recanalization status.

**Conclusion:** The NEMMI score is a quick and simple rating tool of early ischemic changes on CT and could serve as an important surrogate marker for developing malignant edema. Its diagnostic accuracy was similar to CTP and clinical parameters.

## Background

Stroke due to large vessel occlusion (LVO) is one of the major global causes of disability and death (1). Although endovascular thrombectomy has significantly improved clinical outcome, in particular after middle cerebral artery (MCA) occlusion, some patients nevertheless develop progressive cerebral edema with mass effect and transtentorial herniation, defined as malignant middle cerebral artery infarction (MMI) (2, 3). Rapid identification of patients at risk of life-threatening edema is important at an early stage of the clinical workflow in order to consider more rigorous monitoring of patients and, ultimately, triage for decompressive hemicraniectomy (4). A recent meta-analysis of multiple randomized trials for decompressive hemicraniectomy in LVO stroke with a total of 338 patients demonstrated a significant reduction of mortality after decompressive surgery by 39% compared to best medical treatment, and an increase in the number of patients with only mild to moderate disability (modified Rankin scale 2–3) by 13% (5). Cerebral edema due to ischemic stroke is considered to reach its maximum extent within 2–5 days after the initial vessel occlusion (4). In the case of malignant infarction, this usually comes with severe clinical deterioration. With high probability of already manifest brain tissue damage secondary to increased intracranial pressure at time of herniation, patients at risk of MMI should be identified at an earlier timepoint in order to benefit as much as possible from invasive therapy (6, 7). Clinical independent predictors of MMI have been widely discussed in the literature and include elevated levels on the National Institute of Health Stroke Scale (NIHSS), history of arterial hypertension, female sex, congestive heart failure and younger age (8–11). Radiological predictors include a lower ASPECTS with hypodense ischemic changes on non-enhanced CT (NECT) secondary to increased net water uptake (NWU) (12–14). Nevertheless, these areas of hypodensity in NECT are often subtle and depend on time passed since stroke onset and other factors, in particular the presence of intracranial collaterals and core infarct volume (15). CT Perfusion (CTP) with measurement of CBV and permeability may further facilitate early detection of malignant edema, but requires multimodal imaging (13, 16, 17). In addition, higher clot burden, more proximal thrombus location, and poor intracranial collaterals have been described as predictors for MMI in CT-angiography (13). Yet, simple and widely feasible approaches to predict MMI at an early stage are sparse.

We hypothesized, that in patients with acute LVO, a scoring system based on simple measurement of the maximum anterior-posterior and mediolateral diameter of hypodense ischemic changes (AP+ML) in admission NECT, multiplied by a visually rated hypoattenuation factor of the hypodense lesion is associated with MMI (NEMMI score). The aim of this study was to assess the diagnostic ability of the NEMMI score in comparison to other common predictors of a malignant course after LVO. For this purpose, performance of the NEMMI score was tested against CTP-derived core lesion volume, and baseline clinical parameters.

## Materials and Methods

### Patients

Ischemic stroke patients with LVO of the MCA territory admitted between January 2014 and July 2016 at the University Medical Center Hamburg-Eppendorf, Germany (*n* = 109 patients) were retrospectively screened. As only anonymized data were registered, no informed consent was necessary in accordance with the ethical review board approval from the Ethics Committee of the Hamburg Chamber of Physicians (Hamburg, Germany).

The data on which the findings of this study are based are available from the corresponding author on reasonable request.

Patient inclusion criteria for this study were as follows: (1) Acute ischemic MCA stroke secondary to LVO confirmed by multimodal CT within 6 h from symptom onset (non-enhanced CT [NECT], CT angiography, and CT perfusion [CTP]); (2) Visually evident early infarct lesion with hypoattenuation in admission NECT and/or lesion in CTP with reduced cerebral blood volume; (3) Follow-up CT 24–48 h after symptom onset; (4) Documented NIHSS score on admission; (5) Absence of intracranial hemorrhage and/or presence of old infarct lesions in admission NECT. Baseline and follow-up clinical characteristics and demographic information were extracted from the medical records, including use of mechanical recanalization and/or need for decompressive hemicraniectomy.

### Definition of Malignant Infarction

Presence of malignant middle cerebral artery infarction (MMI) was defined as space-occupying infarct (>1/2 affected MCA territory) in follow-up CT at 24–48 h after admission with neurological signs of transtentorial/subfalcine herniation requiring decompressive hemicraniectomy or subsequent death secondary to edematous mass effect (12, 17, 18).

### Imaging

Patients received a multimodal stroke imaging protocol at admission with NECT, CT angiography and CTP on an iCT 256 (Philips Healthcare, Best, the Netherlands) or SOMATOM force (Siemens Healthcare, Erlangen, Germany) scanner. NECT: Collimation 64 × 0.6 (force: 96 × 0.6), pitch 0.297 (force: 0.55), rotation time 0.4 s (force: 1), field of view 270 mm, tube voltage 120 kV (force: 100), tube current 300 mA (force: 406), 4.0 mm slice reconstruction. CT angiography: Pitch 0.985 (force: 0.35), rotation time 0.4 s (force: 0.25), field of view 220 mm, tube voltage 120 kV (force: 70–120), 300 mAs (force: 117–200), 2.0 mm slice reconstruction, 5 mm maximum intensity projection reconstruction with 1 mm increment. CTP: Rotation time 0.5 s (force: 0.5), field of view 220 mm, tube voltage 80 kV (force: 70), tube current 140 mAs (force: 170), 5 mm slice reconstruction, slice sampling rate 1.8 s (force 1.5), biphasic injection with 40 mL of highly iodinated contrast medium with 400 (mmol/L)/mL injected with 6 mL/s followed by 40 mL saline chaser bolus. Imaging data sets were assessed for quality with exclusion in case of severe motion artifacts. Raw perfusion data were analyzed on a Siemens workstation using Syngo VPCT Neuro software (Siemens Healthcare, Erlangen, Germany) or on a Philips workstation using IntelliSpace Portal software (Philips Healthcare, Best, the Netherlands).

### Assessment of NEMMI Score

Ratings were performed retrospectively on NECT images acquired not later than 6 h after symptom onset by two experienced radiologists (>5 years clinical practice). The raters were blinded to specific patient data (i.e., age, subsequent evolution of malignant infarction). Scans with early hypoattenuated infarct lesions were than selected for assessment of the NEMMI score. The ratings were assessed for interrater reliability. Subsequently, a consensus reading of the cases was performed and only consensus measurements used for analyses.

**Figure 1:**
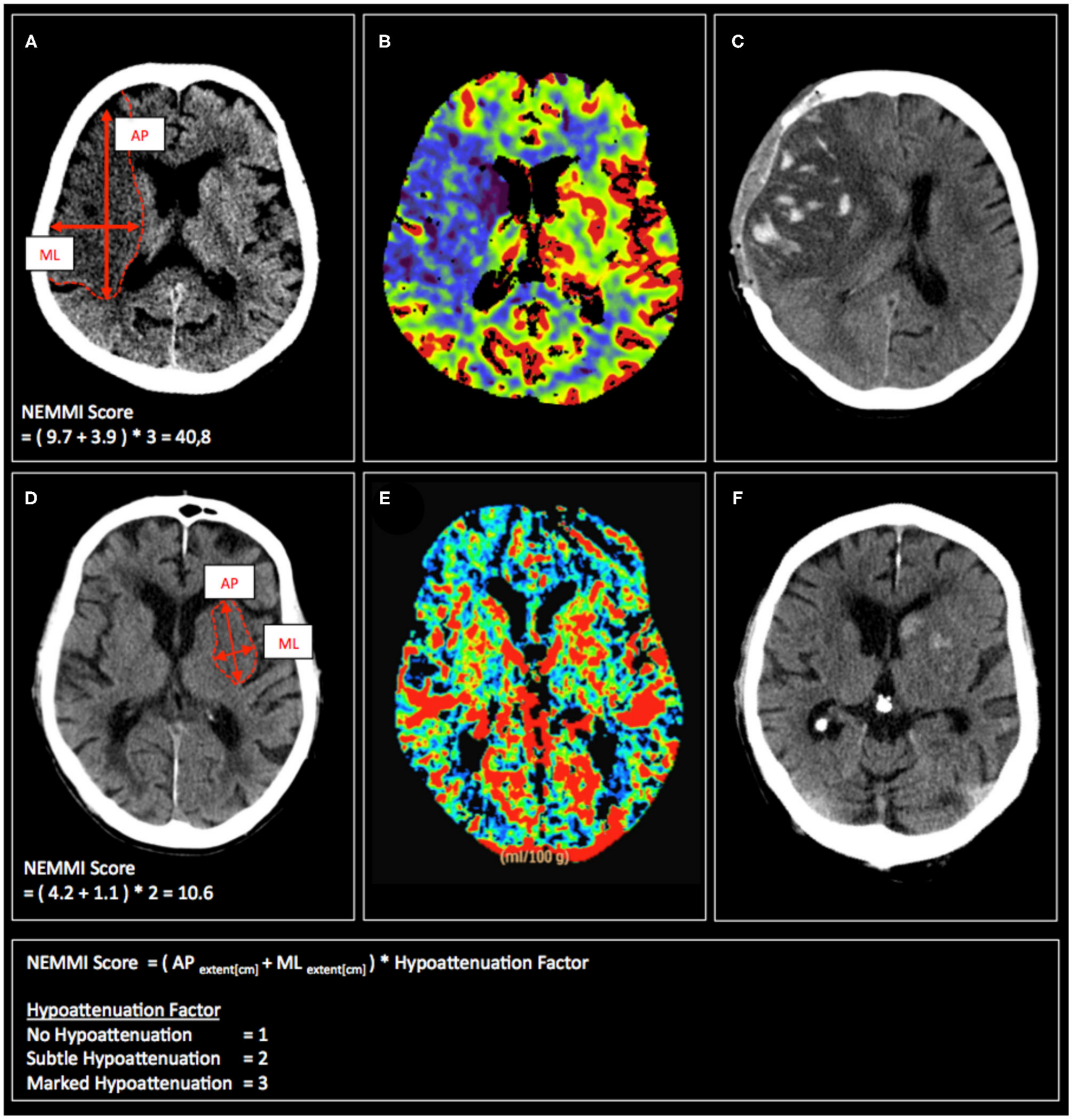
Exemplary admission CT scans **(A,D)**, CBV maps **(B,E)**, and follow-up CT scans **(C,F)** of patients with acute MCA occlusion, who subsequently develop malignant infarction (upper row) or have a non-malignant clinical course (lower row). Calculation of the respective NEMMI scores as the sum of the maximum AP and ML early lesion diameter multiplicated by the visual hypoattenuation factor is depicted in **(A,D)**.

**Figure 2:**
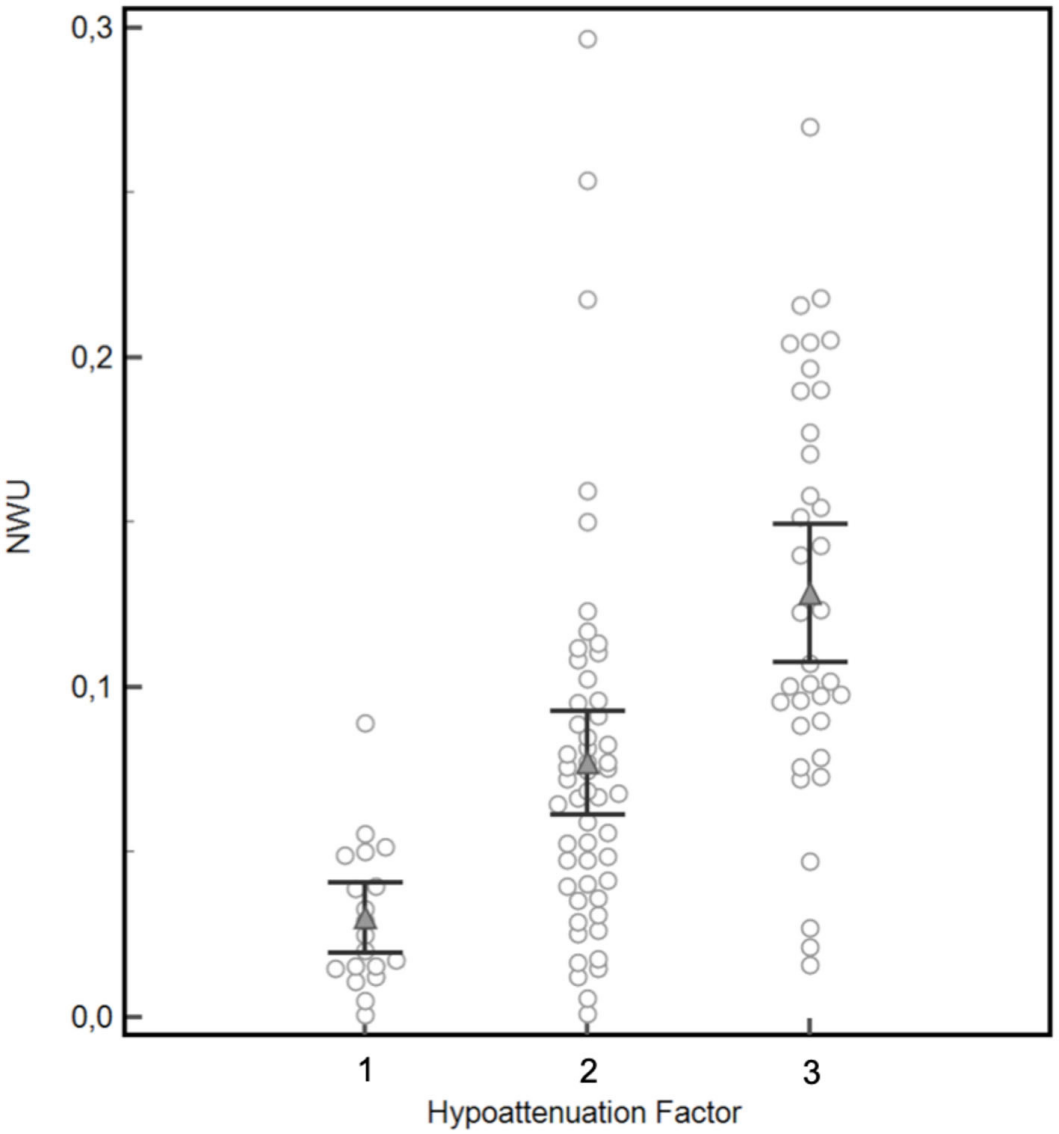
Validation of the visually rated hypoattenuation factor by semi-automatic quantification of net water uptake (NWU). Mean NWU differed significantly between the three patients groups classified for hypoattenuation factor 1 (mean NWU 3.0%), 2 (mean NWU 7.7%), and 3 (mean NWU 12.9%), black brackets indicate 95%CI, *p* = 0.0001 for factor 1 vs. factor 2, *p* < 0.0001 for factor 2 vs. factor 3.

The assessment of the NEMMI score was conducted in the following way: In the axial slice with the most prominent hypoattenuation, the maximal anterior-posterior (AP) and medio-lateral (ML) extension of the early lesion was measured in centimeters [cm] with the measurement tool provided by the image viewer of the hospital radiological information system (Centricity RIS-i 6.0, General Electric, Boston, MA, USA). In order to take into account varying hypoattenuation of the core lesion, the sum of the maximum lesion dimension (AP+ML [cm]) was then multiplied by a hypoattenuation factor of the hypodense lesion (3-point visual estimate of the hypodensity: No hypodensity = 1; Subtle hypodensity = 2; Clearly demarcated hypodensity = 3; Figure 1). In order to validate the visually assessed hypoattenuation factor, the gold standard of net water uptake (NWU) was assessed through a standardized image post-processing protocol according to Broocks et al. (12) and compared to the initially rated hypoattenuation factor (Figure 2).

### Statistical Analysis

Absolute and relative frequencies for all patient characteristics were determined, separately for patients with and without MMI. A *t*-test was performed to compare ordinal data with normal distribution, while a Mann–Whitney *U*-test was used otherwise. A *p*-value of <0.05 was regarded as statistically significant. Medians are illustrated with the respective interquartile ranges (IQR), means with the respective standard deviation (SD).

The Spearman's rank correlation coefficient was measured to assess the correlation between the visually rated hypoattenuation factor and the conventionally quantified NWU.

The NEMMI score was compared with the most relevant alternative clinical and imaging variables to predict MMI (admission NIHSS score, age, CTP core lesion volume, and recanalization status). Ischemic core volume was defined using absolute cerebral blood volume (CBV) with a threshold at 2.0 ml × 100 g^−1^, as described by Wintermark et al. (19). Univariable receiver operating characteristic (ROC) curves with the corresponding area under the curve (AUC) were determined to compare the ability of NEMMI and alternative variables at admission to discriminate between patients with MMI and non-malignant MCA territory infarctions. Inter-rater agreement was quantified using intra-class correlation coefficients (ICC).

In order to investigate the independent multivariable contribution of NEMMI and other variables to predict MMI, multivariable logistic regression analyses were performed presenting odds ratio (OR) estimates along with 95% confidence intervals using backwards selection of the independent variables (age, NIHSS, time from onset to imaging, baseline ischemic core volume, NEMMI, and recanalization status).

The open-source statistical software R (The R Foundation) was used for statistical analysis, and the R package ggplot2 for visualization. Receiver operating characteristic analyses were calculated with MedCalc version 12.7 (MedCalc Software, Ostend, Belgium).

## Results

A total of 109 patients fulfilled the inclusion criteria. Of these, 94 (86.2%) had a non-malignant course of disease, while 15 (13.8%) suffered from MMI with mass herniation. Patient characteristics are depicted in Table 1. Median NIHSS on admission (Non-MMI 16 [IQR 12–19]; MMI: 17 [IQR 16–18]; *p* = 0.158) and mean time from symptom onset to hospital admission (Non-MMI 3.3 h [SD 1.8]; MMI: 2.6 [SD 1]; *p* = 0.165) were similar in both groups. Ischemic core volumes showed high variations in non-MMI and MMI patients, with a mean volume of 16.9 mL (SD 17.6) and 50.1 mL (SD 37.7; *p* < 0.005), respectively. Follow-up infarct volume up to 48 h after admission was significantly elevated in patients with malignant clinical course (204.3 mL [SD 57.1] vs. 48.6 mL [SD 51.3]; *p* < 0.00001). The majority of patients in both groups received intravenous lysis (77.6 and 73.3%; *p* = 0.715). Endovascular thrombectomy was more frequent in the non-malignant group (96.8 vs. 73.3%; *p* < 0.0007), with also significant higher proportion of successful mechanical recanalization (TICI 2b/3: 68.1 vs. 18.2%; *p* < 0.002).

**Table 1:** Patient characteristics stratified by malignant and non-malignant infarcts.

**Patient characteristics**	**Non-malignant**	**Malignant**
Subjects, *n* (%)	94 (86.2)	15 (13.8)
Age in years, mean (SD)	68.6 (14.8)	56.4 (12)
Female sex, *n* (%)	48 (51.1)	6 (40)
Admission NIHSS, median (IQR)	16 (12–19)	17 (16–18)
Time from onset to admission, mean h (SD)	3.3 (1.8)	2.6 (1)
Volume of early infarct (core volume), mean mL (SD)	16.9 (17.6)	50.1 (37.7)
Follow-up infarct volume, mean mL (SD)	48.6 (51.3)	204.3 (57.1)
Intravenous lysis, *n* (%)	73 (77.6)	11 (73.3)
Endovascular thrombectomy, *n* (%)	91 (96.8)	11 (73.3)
If thrombectomy performed: TICI 2b/3, *n* (%)	62 (68.1)	2 (18.2)
Anterior-posterior (AP) diameter of early lesion, median cm (IQR)	3.1 (1.8–4)	3.9 (3.1–6.3)
Mediolateral (ML) diameter of early lesion, median cm (IQR)	1.6 (1–2)	2.3 (1.8–3.7)
Hypoattenuation factor, median (IQR)	2 (1–2)	3 (2–3)
NEMMI score, median (IQR)	7.7 (3.9–11.2)	13.6 (11.6–31.1)

*NIHSS, National Institute of Health Stroke Scale; SD, standard deviation; IQR, interquartile range; TICI, thrombolysis in cerebral infarction; NEMMI, non-enhanced malignant media infarction*.

Inter-rater agreement was tested separately for the hypoattenuation factor, and both diameters (AP and ML). The intraclass correlation coefficient showing the reliability of averages of κ ratings was 0.88 (95%CI: 0.78–0.93) for the hypoattenuation factor, and 0.91 (95%CI: 0.84–0.95)/0.88 (95%CI: 0.79–0.94) for the AP and ML diameter, respectively.

Measurement of the median maximum lesion diameter on admission NECT was similar in both groups (AP: Non-MMI 3.1 cm [IQR 1.8–4]; MMI 3.9 cm [IQR 3.1–6.3]; *p* = 0.144/ML: Non-MMI 1.6 cm [IQR 1–2]; MMI 2.3 cm [IQR 1.8–3.7]; *p* = 0.082).

Mean NWU differed significantly between the three patients groups classified for hypoattenuation factor 1 (mean NWU 3.0%), 2 (mean NWU 7.7%), and 3 (mean NWU 12.9%), *p* = 0.0001 for factor 1 vs. factor 2, *p* < 0.0001 for factor 2 vs. factor 3 (Figure 2). Accordingly, a significant positive correlation was demonstrated for a visually assessed elevated hypoattenuation factor and increased NWU (Spearmans rho = 0.6, *p* < 0.0001, 95%CI of rho: 0.50–0.71).

**Figure 3:**
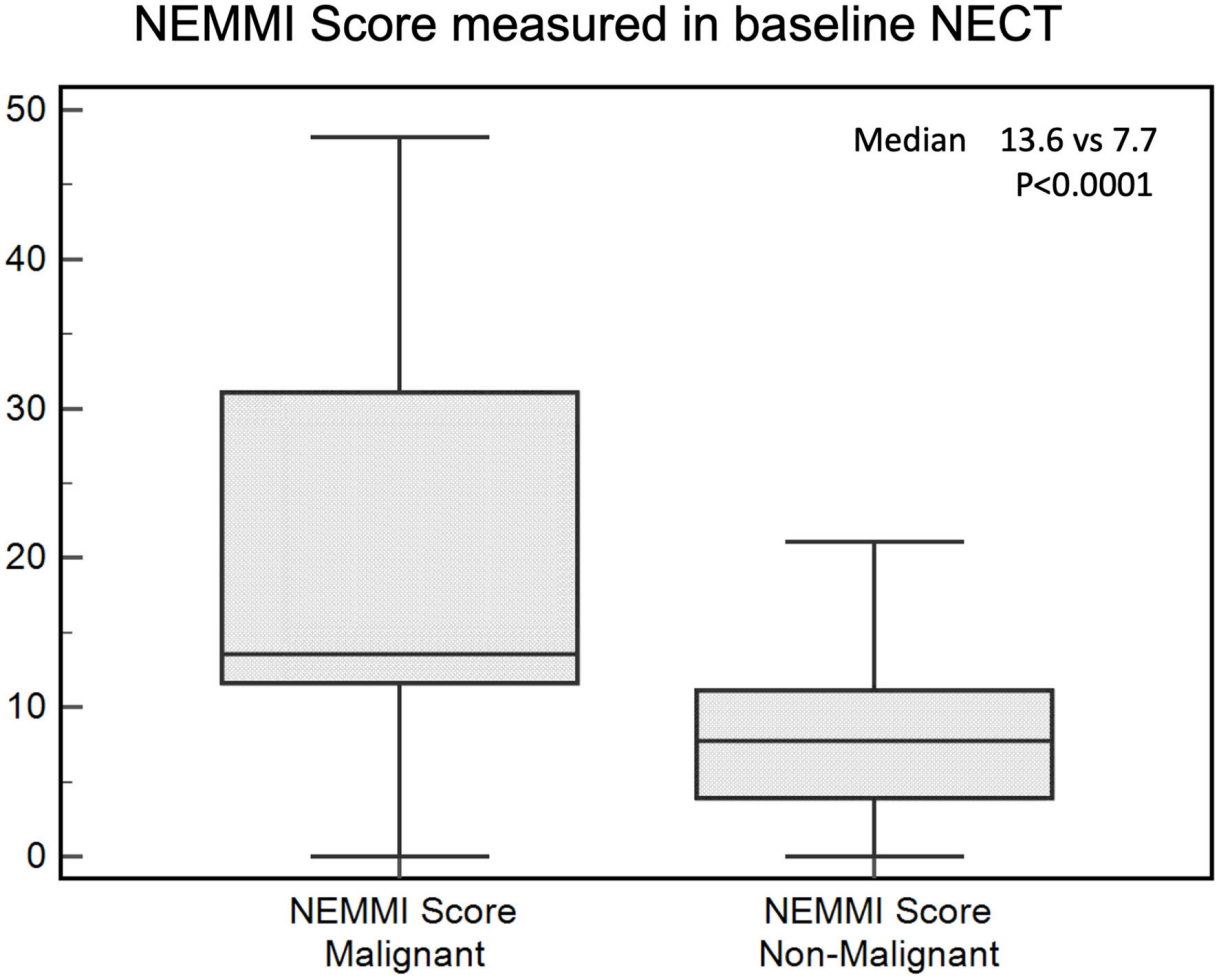
NEMMI scores assessed for patients with subsequent malignant (MMI) and non-malignant course. Medians are illustrated as boxplots with the corresponding interquartile ranges. The NEMMI score was significantly elevated in patients with subsequent MMI.

The median NEMMI score was significantly elevated in patients developing subsequent MMI during their clinical course (7.7 [IQR 3.9–11.2] vs. 13.6 [IQR 11.6–31.1]; *p* < 0.0001; Figure 3).

**Figure 4:**
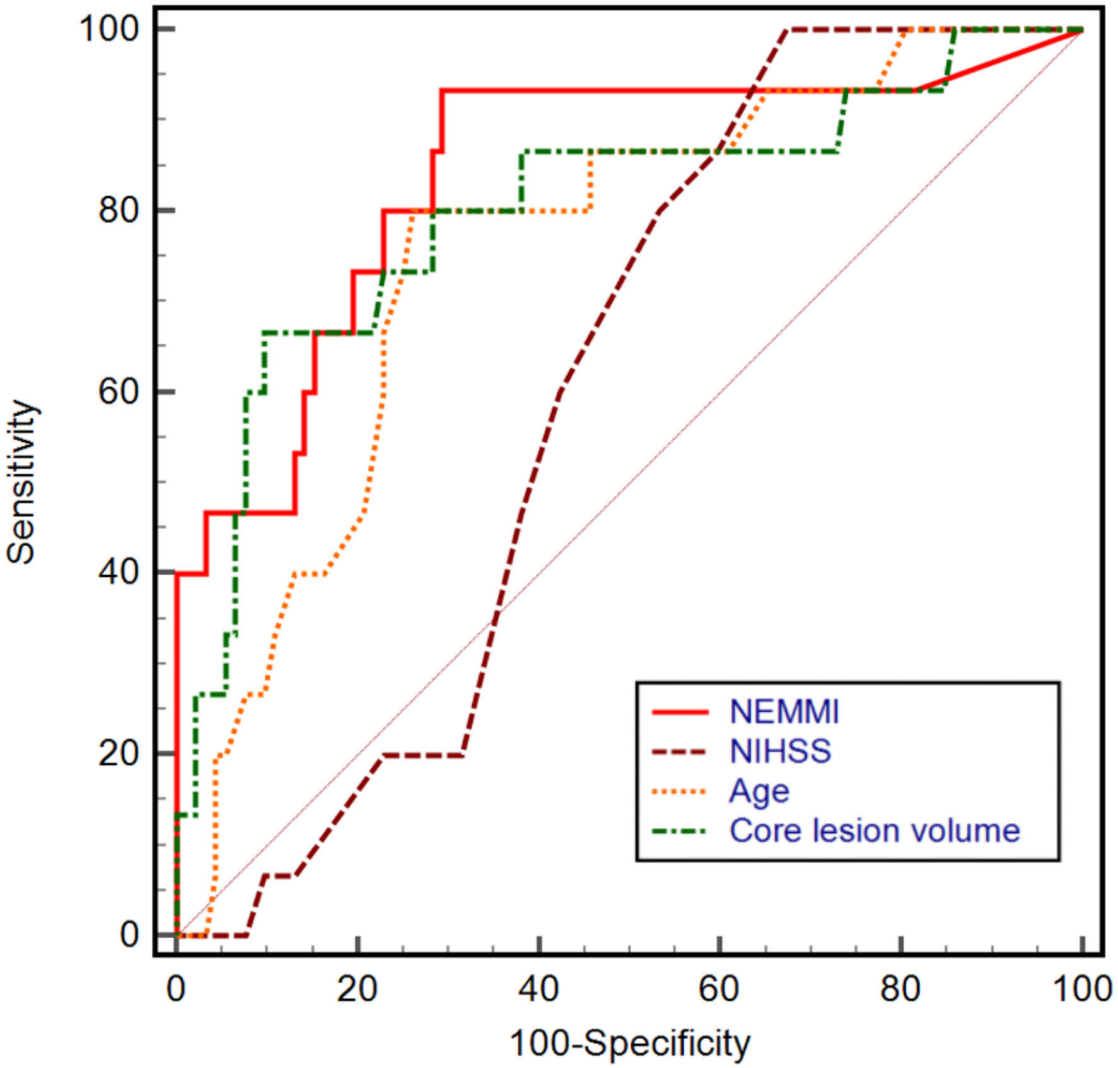
Univariable receiver operating characteristic (ROC) curves with the corresponding area under the curve (AUC) of NEMMI score, admission National Institute of Health Stroke Scale (NIHSS) score, age, and core lesion volume in CT-perfusion. The power to discriminate between subsequent non-malignant and malignant infarction was highest for a NEMMI score >10.5 (AUC: 0.84, sensitivity: 93.3%, specificity: 70.7%, Youden J: 0.64), followed by core lesion volume (AUC: 0.80), age (AUC: 0.76), and NIHSS (AUC: 0.61).

Based on univariable ROC curve analysis, a NEMMI score >10.5 identified MMI with high discriminative power (AUC: 0.84; sensitivity: 93.3%; specificity: 70.7%; Youden J: 0.64), which was higher compared to age (AUC: 0.76), NIHSS (AUC: 0.61), or core lesion volume (AUC: 0.80; Figure 4).

**Figure 5:**
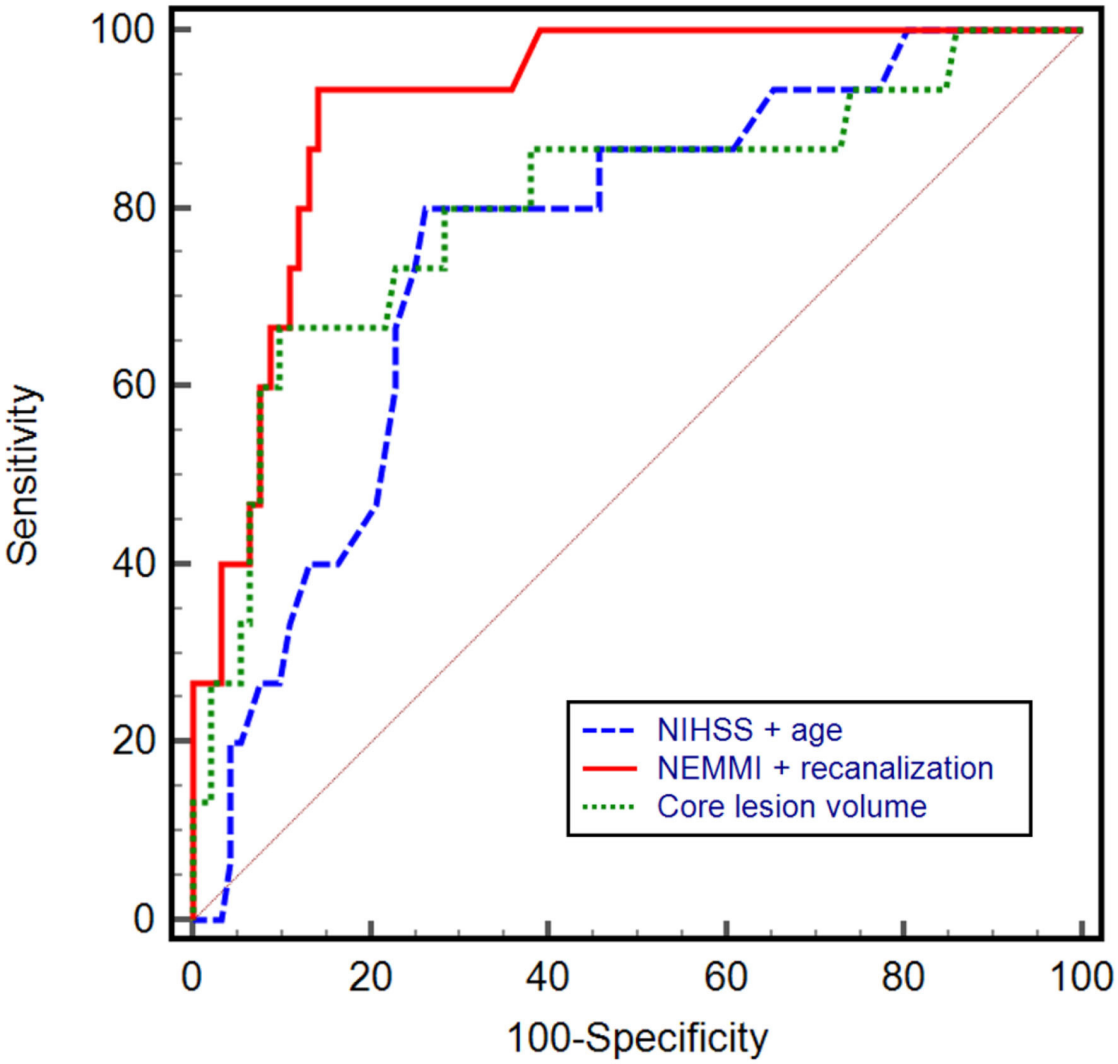
Receiver operating characteristic (ROC) curves with the corresponding area under the curve (AUC) of different prediction models. A combined model including the NEMMI score and recanalization status demonstrated the highest diagnostic power (AUC: 0.92, 95%CI: 0.85–0.96), compared to the clinical model NIHSS + age (AUC 0.78; 95% CI 0.69–0.85) and core lesion volume (AUC 0.80; 95% CI 0.72–0.87). Not shown in the graph for reason of clarity: NEMMI score + age + recanalization (AUC 0.92; 95%CI 0.86–0.97); NIHSS + age + recanalization (AUC 0.89; 95%CI 0.81–0.94), NIHSS + recanalization (AUC 0.84; 95%CI 0.76–0.9).

In multivariable logistic regression analysis, NEMMI score was significantly associated with MMI (odds ratio: 1.33; 95%CI: 1.13–1.56; *p* < 0.001). Further independent variables that were significantly associated with MMI were recanalization status (OR: 0.04; 95%CI: 0.003–0.39; *p* = 0.006), and time from onset to imaging (OR: 0.22; 95%CI: 0.06–0.78; *p* = 0.02; see Table 2). A logistic regression model consisting of the NEMMI score and recanalization showed the highest diagnostic power to predict MMI (AUC: 0.92; 95%CI: 0.85–0.96), equivalent to the model NEMMI score + age + recanalization (AUC 0.92; 95%CI 0.86–0.97), and compared to the clinical models NIHSS + age + recanalization (AUC 0.89; 95%CI 0.81–0.94), NIHSS + recanalization (AUC 0.84; 95%CI 0.76–0.9), NIHSS + age (AUC 0.78; 95% CI 0.69–0.85), and core lesion volume (AUC 0.80; 95% CI 0.72–0.87; Figure 5).

**Table 2:** Multivariable logistic regression analysis for risk analysis of MMI.

	**Odds ratio**	**95%CI**	***P*-value**
Age	0.93	0.87–1	0.055
NIHSS at admission	1.15	0.95–1.39	0.15
NEMMI	1.33	1.13–1.56	0.001
Time from onset to imaging	0.22	0.06–0.79	0.02
Core lesion volume	0.99	0.95–1.04	0.71
Successful recanalization	0.04	0–0.39	0.006

*NIHSS, National Institute of Health Stroke Scale; CI, confidence interval; NEMMI, non-enhanced malignant media infarction*.

## Discussion

Early triage of patients at high risk of malignant infarction for decompressive hemicraniectomy is a clinical challenge and commonly subject of interdisciplinary discussion. The major predicament is to either wait until neurological deterioration paralleled by radiological findings of mass effect and therefore secondary tissue damage are present or to intervene preemptively with the associated risks of an invasive, potentially non-justified treatment and hence secondary worse outcome. Our study demonstrates the high predictive value of the NEMMI score in admission NECT to identify patients at risk of subsequent malignant infarction with clinical deterioration, need of decompressive craniectomy or letal outcome secondary to mass effect of the edematous tissue.

Other studies suggested accurate prediction of MMI on the basis of multimodal stroke imaging at admission (12, 17, 20, 21). However, these prediction models rely on parameters retrieved from image post-processing. Although this can be achieved semi-automatically, the process is still time consuming, i.e., requires mostly manual segmentation of the cerebrospinal fluid compartment and CBV lesion volume, or quantification of parenchymal net water uptake (12, 17, 22). The latter was demonstrated to be highly associated with a malignant course, but quick assessment is not feasible as it requires calculation of mean densities. An alternative MMI risk prediction model is the EDEMA (Enhanced Detection of Edema in Malignant Anterior Circulation Stroke) score, which is based on clinical and imaging variables within the first 24 h after stroke onset and has recently been externally validated in a Chinese patient cohort (20, 21). Nevertheless, the score cannot be assessed upon admission, as it incorporates features only available after admission CT, i.e., reperfusion therapy, blood glucose and presence of midline shift within 1 day after symptom onset. In contrast, the assessment of the NEMMI score is based on simple measurement of the early lesion extent and visual quantification of the lesion hypodensity in one axial slice of the initial NECT. This can be achieved bedside in the emergency department with conventional radiological image viewers, without the necessity of obtaining additional clinical and laboratory parameters.

This allows consideration of a potential surgical decompressive treatment at the same time as other critical decisions are made by the treating physicians, in particular triage for mechanical thrombectomy or intravenous lysis.

To our knowledge, this study is the first to describe a simple imaging-based method to predict MMI at an early stage that does not require extensive post-processing, or time-consuming measurements.

The ASPECT (Alberta Stroke Program Early CT) score is a well-established imaging parameter for hypodense lesion involvement in acute MCA occlusion (23–25). Nevertheless, since the subterritorial regions assessed by the ASPECT score are each of different size but still count equally in terms of cumulative region involvement, it is not a quantitative score with respect to volume of affected tissue. In contrast, the proposed NEMMI score incorporates direct lesion diameters independent of the subterritorial location. As severity of brain swelling with mass effect is directly linked to baseline volume of edematous brain tissue, a score based on lesion dimensions rather than region involvement may be of advantage for MMI prediction (12, 23, 26).

The NEMMI score showed a high diagnostic power to predict a malignant infarction (AUC: 0.84), similar to CTP-derived core lesion volume (AUC: 0.76), or clinical parameters (NIHSS, AUC: 0.61; age, AUC: 0.76). If validated on a larger patient cohort, this would support rapid risk-stratification of early decompressive therapy in patients with MCA occlusion independent of the availability of advanced stroke imaging. Furthermore, the NEMMI score addresses a common problem in detection of early ischemic changes on admission NECT: subtleness of the early lesion in relation to the extent of the lesion. While lesions may be large in size, the hypodensity may only be subtle and vice versa. The intensity of the edema is incorporated by grading the hypoattenuation of the early lesion on a quasi-quantitative scale (3-point visual grading of the hypodense lesion, no/subtle/clear, 1–3). Other studies have demonstrated hypodense NECT changes in more than 50% of the MCA territory as independent predictor of MMI (8, 9). This method requires volumetry of the ischemic changes and does not take into account the degree of the density changes. Nevertheless, further studies are necessary to compare different predictors of MMI in NECT with respect to predictive quality and acquisition speed.

In this study, core lesion volume was not associated with MMI in multivariable logistic regression analysis (Table 2), which is in accordance with recent studies observing that core lesion volume is not associated with functional outcome in low ASPECTS patients (27). Second, core lesion volume did not modify the effect of mechanical thrombectomy according to a recent meta-analysis (28).

We do not suggest that an elevated NEMMI score alone would serve as an indication for decompressive surgery. The score may rather facilitate decision making as it provides information about both lesion extent and progression in an acute clinical setting with high availability, applicability, and acquisition speed.

Further internal and external validation of the NEMMI score is necessary in a large patient cohort. Moreover, future studies should investigate how treatment factors such as successful or failed recanalization impact the predictive power of the score (29).

A major limitation of the current study and the proposed NEMMI score is the potentially compromised interrater reliability of the visually assessed hypoattenuation factor. The visual differentiation between a subtle or clearly demarcated hypoattenuation depends on various factors, such as type of scanner, image quality, slice thickness etc. An exact range of intensities (i.e., Hounsfield units) is difficult to define and would have to be adjusted for these variables. In contrast, experienced radiologists (in our study >5 years clinical practice) can judge hypoattenuations in scans acquired on a familiar scanner and with a standardized imaging protocol in a timely manner with acceptable objectiveness. Visual ratings in our study correlated significantly with NWU as semi-automatic standard measurement of ionic/vasogenic tissue edema and hence tissue hypoattenuation (12). While also interrater reliability was robust for both the measurement of the lesions diameters and the hypoattenuation factor, future studies aiming to validate the score will have to quantify this effect among various raters. Important in this context will be the simultaneous semi-automatic assessment of the NWU as independent marker of lesion hypodensity in order to either calibrate the visual grading or to serve as an objective parameter for comparison (30).

Due to strict inclusion criteria and narrow definition of malignant infarction the current study relies on a small patient sample and has therefore limited generalizability. Validation studies on a larger patient cohort will have to focus on the exact validity of the NEMMI score for prediction of MMI, ideally with definition of thresholds dependent on subsequent endovascular therapy and degree of recanalization. In the pre-thrombectomy era, prevalence of MMI was up to 10% in patients with MCA occlusion and significantly reduced during the era of endovascular thrombectomy (2, 31, 32).

## Conclusion

The NEMMI score is a quick and simple rating tool of early ischemic changes on non-enhanced admission CT without the necessity of multimodal imaging or time-consuming image post-processing. If validated on a larger patient cohort, it may serve as surrogate marker for developing malignant edema and hence facilitate early triage of patients for invasive monitoring or decompressive hemicraniectomy.

## Data Availability Statement

The original contributions presented in the study are included in the article, further inquiries can be directed to the corresponding author.

## Ethics Statement

The studies involving human participants were reviewed and approved by Ethics Committee of the Hamburg Chamber of Physicians (Hamburg, Germany). Written informed consent for participation was not required for this study in accordance with the national legislation and the institutional requirements.

## Author Contributions

MB and GB conceptualized and supervised the study, acquired and analyzed the data, and wrote the manuscript. SB acquired the data. JF and UH analyzed the data and critically reviewed the manuscript. LM, NH, TF, and RM critically reviewed the manuscript. All authors contributed to the article and approved the submitted version.

## Conflict of Interest

JF receives research support from EU, BMBF, BMWi, DFG, Acandis, Medtronic, Microvention, Stryker. He is consultant for Acandis, Codman, Cerenovus, Medtronic, Microvention, Penumbra, Phenox, and Stryker, holds Stock of Tegus Medical and has executive functions at Eppdata (all unrelated). The remaining authors declare that the research was conducted in the absence of any commercial or financial relationships that could be construed as a potential conflict of interest.
